# Altered Immune Response and Implantation Failure in Progesterone-Induced Blocking Factor-Deficient Mice

**DOI:** 10.3389/fimmu.2020.00349

**Published:** 2020-03-11

**Authors:** Timea Csabai, Eva Pallinger, Arpad F. Kovacs, Eva Miko, Zoltan Bognar, Julia Szekeres-Bartho

**Affiliations:** ^1^Department of Medical Biology and Central Electron Microscope Laboratory, Medical School, Pecs University, Pecs, Hungary; ^2^János Szentágothai Research Centre, Pecs University, Pecs, Hungary; ^3^Endocrine Studies, Centre of Excellence, Pecs University, Pecs, Hungary; ^4^MTA-PTE Human Reproduction Research Group, Pecs, Hungary; ^5^Department of Genetics, Cell, and Immunobiology, Semmelweis University, Budapest, Hungary; ^6^Department of Medical Microbiology and Immunology, Medical School, Pecs University, Pecs, Hungary

**Keywords:** PIBF, decidual NK cells, T cell activation, B cells, implantation

## Abstract

Earlier data suggest that progesterone-induced blocking factor (PIBF) is involved in implantation. The present study therefore aims to investigate the consequences of functional PIBF deficiency during the peri-implantation period. CD1 female mice were injected intraperitoneally with 2 μg anti-PIBF monoclonal antibody on days 1.5 and 4.5 of pregnancy. The number of implantation sites and resorption rates were recorded on day 10.5. PIBF+ decidual NK cells and B cells were detected by immunohistochemistry or immunofluorescence. Decidual and peripheral NK activity was assessed by flow cytometry. A prime PCR array was used for determining the differential expression of genes involved in lymphocyte activation and Th1 or Th2 differentiation in CD4+ and CD8+ spleen cells from pregnant anti-PIBF-treated and control mice. Anti-PIBF treatment in the peri-implantation period resulted in impaired implantation and increased resorption rates in later pregnancy. The number of PIBF+ decidual NK cells decreased, while both decidual and peripheral NK activity increased in the anti-PIBF-treated mice. B cells were absent from the resorbed deciduas of anti-PIBF-treated mice. The genes implicated in T cell activation were significantly downregulated in CD4+ and increased in CD8+ of the anti-PIBF-treated animals. The gene for IL-4 was significantly downregulated in CD4+ cells while that of IL-12A was upregulated in CD8+ cells of anti-PIBF-treated animals. These data suggest that the lack of PIBF results in an impaired T cell activation, together with Th1 differentiation and increased NK activity, resulting in implantation failure.

## Introduction

The success of embryo implantation depends on embryo quality as well as on the receptivity of the maternal endometrium. The process starts with the attachment of the embryo to the endometrial epithelium (1–6), followed by invasion to the decidua. Progesterone plays a central role in this process (4, 6) via the nuclear progesterone receptor (PR) isoforms, PRA and PRB (7, 8). Studies on PR knockout mice revealed that PRA is required for endometrial receptivity and decidualization (9), and consequently, PRA-deficient mice are infertile (10, 11).

The progesterone-induced blocking factor (PIBF) is a progesterone-induced mediator which conveys some of the immunological effects of progesterone. The *Pibf1* gene contains a progesterone response element (12), which is activated following the engagement of PRA in the mouse uterus (13).

Earlier data suggest that PIBF is required for the establishment and maintenance of pregnancy, both in humans and mice. In the sera of pregnant women, PIBF concentrations increase throughout gestation and drop before labor (14). During spontaneous miscarriage or preterm delivery, serum PIBF concentrations fall below the normal levels (15). Anti-PIBF treatment or anti-progesterone treatment of pregnant mice results in increased resorption rates, together with an inversion of the Th1/Th2 cytokine balance (16). The latter is due to the fact that PIBF induces an increased synthesis of Th2 cytokines both *in vitro* (17) and *in vivo* (18). Recent data show that PIBF plays a role in implantation in mice (13).

The decidual transformation of endometrial stromal cells is a prerequisite for a successful implantation. Ablation of PRA but not PRB expression in mice results in a uterine phenotype similar to PRKO, indicating that PRA is the major isoform involved in the regulation of uterine receptivity and decidualization in the mouse (19). It is important to point out that, in humans, PRB is also involved in decidualization (20).

In our hands, during a 6-day culture, PIBF induced the decidual transformation of mouse endometrial stromal cells (13). Furthermore, in the mouse endometrium, PIBF expression significantly increased during the implantation window (13).

The immunological effects of PIBF play an important role in establishing a favorable immunological milieu for the developing fetus.

In spite of the presence of perforin and granzyme in their cytoplasmic granules, the decidual NK cells are weakly cytotoxic. High decidual NK activity might damage the fetus and result in a failed pregnancy. PIBF inhibits the degranulation of NK cells (21). Recently we demonstrated a high number of mouse decidual NK cells that contained PIBF in their cytoplasmic granules, suggesting that the local presence of PIBF might be a factor in the low decidual NK activity (22).

In the present study, we aimed to investigate the consequences of anti-PIBF treatment of pregnant mice during the peri-implantation period on reproductive performance as well as the underlying mechanisms.

## Materials and Methods

### Treatment of Mice

Eight- to 12-week-old CD1 female mice (Charles River, Germany) were caged overnight with CD1 males in an environment controlled for temperature, humidity, and light. Sighting of the vaginal plug was considered as 0.5 day of pregnancy.

Females were injected intraperitoneally with 2 μg of anti-PIBF monoclonal antibody (14) on days 1.5 and 4.5 of pregnancy. The control mice were injected with 100 μl of PBS, among the same conditions. On day 10.5 of pregnancy, the mice were sacrificed, the number of implantation sites as well as resorption rates was recorded, and spleens and deciduas were removed for lymphocyte isolation.

All procedures were approved by the Animal Care Committee of the University of Pecs.

### Isolation of Decidual and Spleen Lymphocytes

Isolated mouse deciduas were minced with scissors and incubated for 30 min with 10 ml (1 mg/ml) of collagenase (collagenase from *Clostridium histolyticum*, type IV, Sigma-Aldrich, USA). The fragments were then passed through a 70-μm mesh and washed with RPMI1640 (Gibco, Life technologies, Scotland).

The pellet was resuspended in 10 ml of fetal calf serum (FCS)-free RPMI and filtered on a 40 μm filter. The cell count was adjusted to 1 × 10^6^/ml in RPMI1640 (Gibco, Life technologies, Scotland) +10% FCS (Gibco, Life Technologies, Scotland) + 1% penicillin/streptomycin (Gibco, Life Technologies, Scotland).

Spleen cells were isolated by passing the spleen through a 100-μm stainless steel mesh and centrifuging for 10 min at 1,000 rpm. The pellet was resuspended in 10 ml RPMI1640, filtered on a 70-μm mesh and further on a 40-μm mesh, washed, and resuspended in RPMI1640. The lymphocytes were isolated on Ficoll-Paque gradient, washed, and resuspended in RPMI1640 + 10% FCS + 1% penicillin/streptomycin.

### Immunohistochemistry

The implantation sites were isolated on day 10.5 of pregnancy, fixed in 6% of buffered formalin, and then embedded in paraffin. Five-micrometer paraffin sections were deparaffinized, rehydrated, and revealed with DAKO Target Retrieval Solution (S1699, Dako, Denmark) at pH 6.0 in a microwave oven. Endogenous peroxidase activity was inhibited with 3% H_2_O_2_, and non-specific antibody binding was blocked with 3% BSA.

The slides were than reacted with 1:25 diluted biotinylated monoclonal anti-PIBF antibody produced in our laboratory (14) or biotinylated mouse IgG2a either for 1 h at room temperature or overnight at 4°C. After incubation, the slides were washed for 3–5 min and reacted with 1:100 diluted streptavidin-horseradish-peroxidase (GE Healthcare, Little Chalfont, United Kingdom) for 30 min in a humidified chamber. The reaction was developed with diamino-benzidine (DAKO, Glostrup, Denmark). The nuclei were counterstained with hematoxylin (DAKO, Glostrup, Denmark) for 3 min, and the slides were mounted.

### Fluorescent Staining

For visualizing of the B cells, the sections were reacted overnight at 4°C with 1:30 diluted rat anti-mouse B220 IgG conjugated with Alexa Fluor 647. The antibody was produced at the Department of Immunology and Biotechnology, University of Pécs, and was provided by Dr. Peter Balogh. The anti-B220 IgG was purified from the supernatant of rat hybridoma RA3-6B2 (obtained from ATCC) using Protein G affinity chromatography. The purified antibody was dialyzed into 0.1 M NaHCO_3_ buffer and conjugated with Alexa Fluor 647 NHS dye (ThermoFisher Scientific) as recommended by the vendor. The conjugated immunoglobulin was separated using Sephadex-G25 size exclusion chromatography.

For identifying of PIBF-positive decidual B cells, the sections were reacted overnight at 4°C with 1:25 diluted FITC-conjugated anti-mouse PIBF antibody, together with the 1:30 diluted Alexa647-conjugated anti-mouse B220. The nuclei were stained with Hoechst33342 (Calbiochem, San Diego, CA, USA) for 5 min, washed, and then mounted with Vectashield mounting medium (Vector Laboratories, Peterborough, United Kingdom) and examined with an Olympus FV-1000 laser scanning confocal system.

### NK Cytotoxicity Test

**Figure 1:**
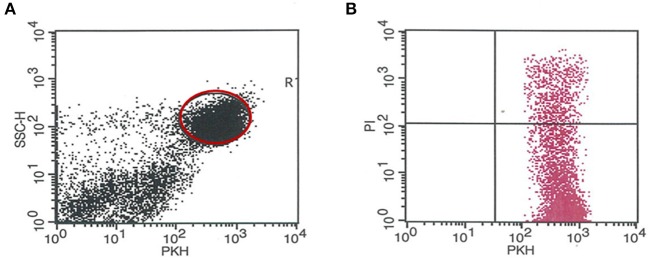
Gating strategy for determining the number of the PKH-67-positive target cells killed (PI+). **(A)** Representative dot plot showing the PKH67 staining of YAC target cells. PKH67-positive cells were gated and were used for further analysis. **(B)** Representative dot plot shows the red fluorescence of PKH67+ YAC cells after PI staining. The percentage of PI+/PKH67+ YAC cells was defined as apoptotic cells.

A flow cytometric assay was used for the determination of the cytotoxic activity of peripheral and decidual natural killer cells from the control and the anti-PIBF-treated pregnant mice. The assay is based on the quantitative and the qualitative flow cytometric analysis of cell damage on a single-cell level. The mouse lymphoma cell line YAC-1 was used as the target cell population. The target cells were pre-stained with PKH-67 (PKH67 Green Fluorescent Cell Linker Midi Kit for General Cell Membrane Labeling, Sigma- Aldrich, USA), a lipophilic dye that stably integrates into the cell membrane without disturbing its surface marker expression. It, thus, permits the distinction between target and effector cells. Freshly isolated peripheral and decidual lymphocytes from pregnant mice served as the effector cells. The target cells and the lymphocytes, at a ratio of 1:12.5, were centrifuged and incubated in RPMI1640 containing 10% FCS and 1% penicillin/streptomycin medium for 4 h at 37°C and in 5% CO_2_. After incubation, the cell mixture was centrifuged and stained with propidium iodide [PI, 50 μg/ml (Sigma-Aldrich, USA)]. PI staining allows the discrimination between death and living target cells. Data analysis is performed first by gating on PKH-67-positive target cells, followed by the analysis of the PI-positive subpopulation killed (gating strategy is shown in Figure 1). The percentage of cytotoxicity in the PKH-67-gated cell population was calculated by subtracting unspecific PI+ positive target cells (spontaneous cell death), measured in appropriate controls without effector cells from the experimental samples. Flow cytometric analysis was performed on a FACS Calibur flow cytometer (BD Immunocytometry Systems, Erembodegen, Belgium) equipped with the CellQuest software program (BD Biosciences, San Diego, CA, USA) for data acquisition and analysis.

### Lymphocyte Activation and Th1 and Th2 Cell Differentiation

A prime PCR array from Bio-Rad was used for determining the markers for T cell activation and Th1 or Th2 differentiation in separated CD4+ and CD8+ spleen cells from anti-PIBF-treated and control pregnant mice. The mice were sacrificed on day 10.5 of pregnancy. The spleens were minced with scissors and passed through a 70-μm cell strainer. The CD4+ and CD8+ T cells were separated from splenic single-cell suspension by magnetic separation with the Mini-MACS system (Miltenyi Biotec Biotechnology Company, USA). Mouse Naive CD8a+ T Cell Isolation Kit (130-096-543) and mouse CD4+ T Cell Isolation Kit (130-104-454) were applied for negative selection, according to the manufacturer's instructions. The collected cells were washed, and the cell count was adjusted to 5 × 10^6^. One hundred thousand separated cells were fluorescently stained with the anti-mouse CD4 or anti-mouse CD8 monoclonal antibodies for checking of the separation efficiency. Cell debris and dead cells were excluded from the analysis based on scatter signals and propidium iodide fluorescence. For the stabilization of RNA, separated CD4+ or CD8+ splenocytes were stored frozen in RNAlater^®^-ICE solution. Total RNA was extracted from the cells with the Qiagen RNeasy Mini Kit (Cat. No. 74104) according to the supplier's protocol. The RNA content of samples was measured with Qubit 4 Fluorometer using the Qubit RNA HS Assay Kit. The expression of 41 genes was determined using a Bio-Rad prime PCR array (Immune response-Th1 and Th2 cell differentiation M384; Bio-Rad Laboratories, Inc., California, USA). Quantitative PCR reactions were carried out on an ABI 7900 real-time PCR instrument according to the manufacturer's instructions.

### Statistical Analysis

Data were analyzed by the Mann–Whitney *U* test and Student's *t* test. *P* ≤ 0.05 was considered as significant.

## Results

### Anti-PIBF Treatment in the Peri-Implantation Period Results in Impaired Implantation and Increased Resorption Rates in Later Pregnancy

**Figure 2:**
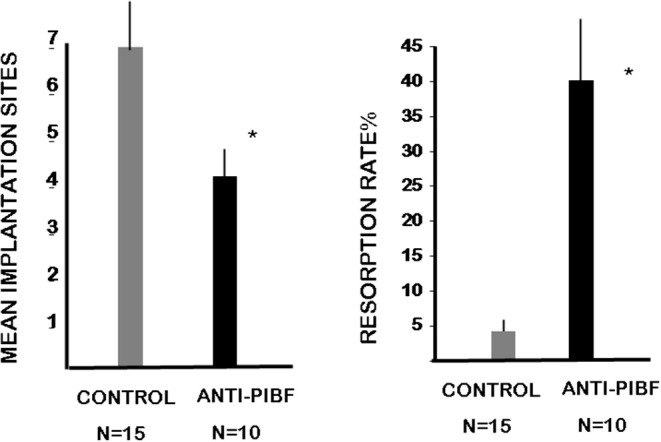
Anti-PIBF treatment of pregnant mice in the peri-implantation period results in decreased implantation and increased resorption rates. CD1 mice were injected with anti-PIBF monoclonal antibody at days 1.5 and 4.5 of pregnancy. The controls were treated with PBS, among the same conditions. The number of implantation sites (1st panel) and resorption rates (2nd panel) were recorded on day 10.5. The implantation sites were significantly lower, while the resorption rates significantly increased in the anti-PIBF-treated mice. The columns represent the mean ± SEM of the results from 10 (anti-PIBF-treated) and 15 (control) mice. ^*^P < 0.05.

The mice were treated with anti-PIBF antibody at days 1.5 and 4.5 of pregnancy to render them functionally PIBF deficient during the implantation window. The mice were sacrificed at day 10.5. This enabled us to record not only the implantation sites but also the resorption rate among the implanted embryos. While the average number of implanted embryos was 6.5 in the controls, in females treated with anti- PIBF antibody in the peri-implantation period, the mean implantation sites decreased to four (Figure 2). The unusually low 2% resorption rate in the control group increased to 40% in the functionally PIBF-deficient mice (Figure 2).

### PIBF+ Large Granulated Cells Are Depleted From the Deciduas of Anti-PIBF-Treated Mice

In an earlier study, we demonstrated a high number of large granulated cells—with a strong PIBF reactivity in the cytoplasmic granules—in the deciduae of day 12.5 pregnant mice. These cells were identified as members of the PAS+ DBA+ uterine NK cell population. PIBF co-localized with perforin in the cytoplasmic granules of the cells (22).

**Figure 3:**
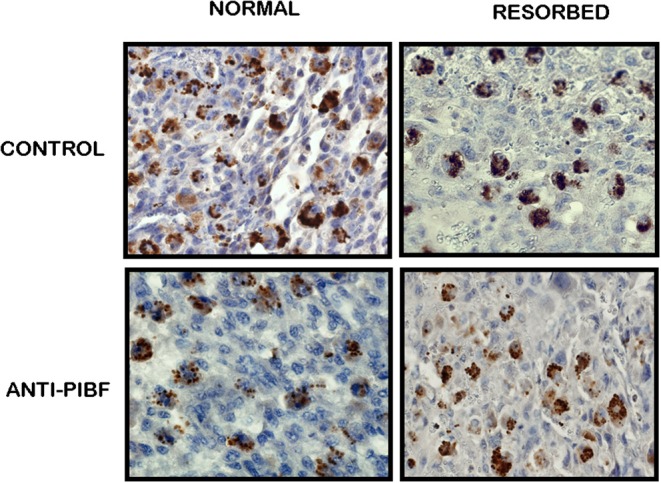
Immunohistochemical analysis of PIBF+ NK cells in normal and resorbed deciduae from anti-PIBF-treated and control mice on day 10.5 of pregnancy (×400).

**Figure 4:**
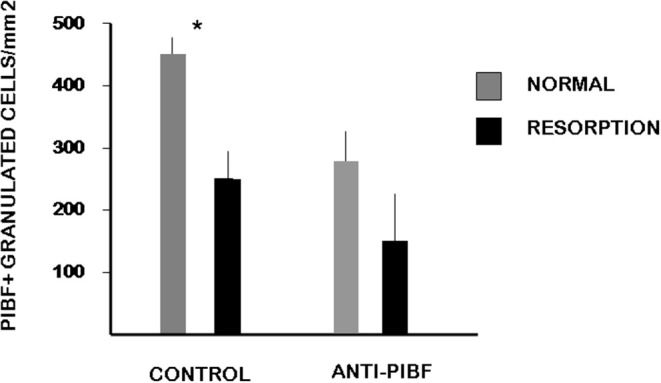
The number of PIBF+ NK cells in deciduas from anti-PIBF-treated and control mice on day 10.5 of pregnancy. Compared to the normal deciduae from the untreated controls, the number of PIBF+ NK cells is significantly lower not only in the deciduae from both the normal and the resorbed fetuses from anti-PIBF-treated animals but also in those of spontaneously resorbed fetuses from control mice. The bars represent the mean ± SEM of 10 independent determinations. ^*^P < 0.05.

In the present study, we found a significantly decreased number of PIBF+ NK cells in the day 10.5 deciduae of resorbed embryos from anti-PIBF-treated mice compared to normal deciduae from untreated mice (Figures 3, 4). The number of PIBF+ granulated cells was also significantly lower in the deciduae of normal embryos from the anti-PIBF-treated mice and in those of spontaneously resorbed embryos from untreated control mice (Figures 3, 4) than in the normal decidua of the untreated mice. These data suggest that the decreased number of PIBF+ decidual lymphocytes was associated with resorption rather than with the lack of functional PIBF.

### Cytotoxic Activity of Decidual and Peripheral NK Cells From Anti-PIBF-Treated and Control Mice

**Figure 5:**
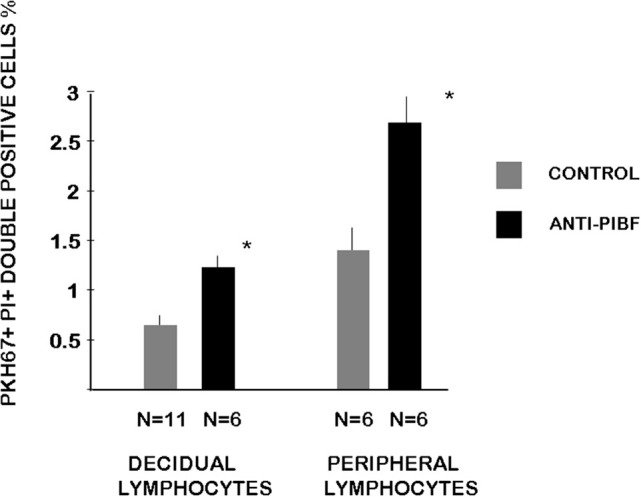
Cytotoxic activity of decidual and peripheral lymphocytes from anti-PIBF-treated and control mice on day 10.5 of pregnancy. The cytotoxic activity of peripheral and decidual NK cells from control and anti-PIBF-treated mice was determined by flow cytometric analysis of target cell damage on a single-cell level. The target cells were labeled with PKH-67 and stained with propidium iodide after 4 h of incubation with the lymphocytes to distinguish apoptotic from non-apoptotic target cells. The bars represent the mean ± SEM of at least six experiments. ^*^P < 0.05.

We determined the cytotoxic activity of decidual lymphocytes and of spleen cells from anti-PIBF-treated and control mice using a flow cytometric method. Both the decidual and the peripheral NK activity of the anti-PIBF-treated mice were significantly (*P* < 0.05) increased compared to those of the controls (Figure 5).

### B Cells Are Depleted From the Deciduas of Anti-PIBF-Treated Embryos

**Figure 6:**
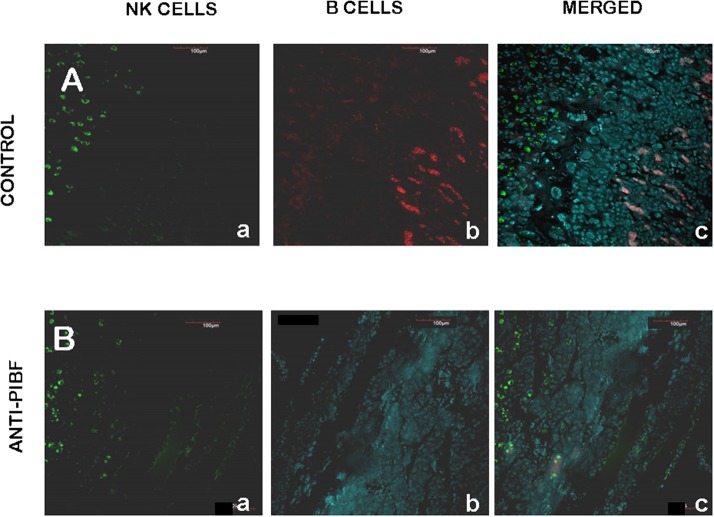
Decidual B cells in control **(A)** and anti-PIBF-treated **(B)** mice. B cells were reacted with rat anti-mouse B220 IgG conjugated with Alexa Fluor 647 (red fluorescence), and NK cells were reacted with fluorescein-conjugated DBA lectin (green fluorescence). **(A)** Decidua of an untreated mouse. NK cells (a,c) are present in the decidua and B cells (b,c) are located at the choriodecidual interface. **(B)** Decidua of anti-PIBF-treated mouse. NK cells (a,c) are present, while B cells (b,c) are absent. (a) NK cells, (b) B cells, and (c) merged (×200).

The endometria of the control animals (Figure 6A) contained decidual NK cells (Figure 6A—a,c) and a discrete layer of B cells (Figure 6A—b,c) at the coriodecidual interface. While the NK cells were still present (Figure 6B—a,c), the B cells disappeared from the deciduas of the resorbed embryos from the anti-PIBF-treated mice (Figure 6B—b,c).

### Functional PIBF Deficiency in the Peri-Implantation Period Results in Impaired CD4+ T Cell Activation and Th1 Type Differentiation

Peripheral Th cell subsets from anti-PIBF-treated and control mice were tested for the differential expression of 48 genes using a prime PCR array. Twelve of these showed a significantly higher or lower expression in the lymphocytes of anti-PIBF-treated mice compared to the controls. When analyzing the results, the differentially expressed genes were assigned to the following groups: (1) genes involved in T cell activation, (2) those involved in Th1 differentiation, and (3) those involved in Th2 differentiation.

**Figure 7:**
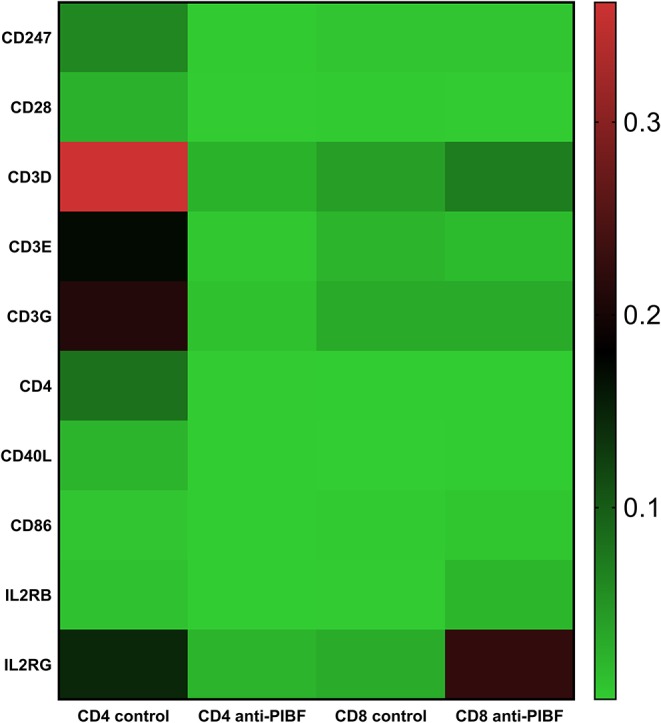
Differential expression of genes implicated in T cell activation in splenic CD4+ and CD8+ T spleen cells of anti-PIBF-treated mice and controls. Heatmap of the T cell activation-related mRNA expression of genes in CD4+ and CD8+ splenocytes of anti-PIBF-treated and control mice. Clear separations are seen between the anti-PIBF-treated and control animals and also between the CD4+ and CD8+ cell types. Members of the CD3 complex and co-stimulatory molecules were downregulated in CD4+ cells and upregulated in CD8+ cells of anti-PIBF-treated mice. All of the results shown were significantly (*P* < 0.05) different from the values of the controls. The expression intensities were scaled on rows (genes) to Z scores to make them weigh equally in the clustering. The colors of the heatmap are mapped linearly to the Z scores (low expression in green and high expression in red).

The genes implicated in T cell activation, e.g., members of the CD3 complex (CD 247, CS3D, CS3E, CS3G, and IL2RG), were significantly downregulated in the CD4+ spleen cells of anti-PIBF-treated mice but significantly increased in the CD8+ cells of the same animals (Figure 7). In the anti-PIBF-treated mice, the beta chain of the IL2R was downregulated in the CD4+ population, while in the CD8 population the alpha and the gamma chain of the IL2R and the IL2 increased. The genes of the co-stimulatory molecules were altered in a similar fashion. Upon anti-PIBF treatment, the genes for CD4, CD28, CD40L, and CD86 were downregulated in the CD4 and upregulated in the CD8 population (Figure 7). These data suggest that the absence of PIBF inhibits the activation of CD4+ cells and facilitates that of CD8+ T cells.

**Figure 8:**
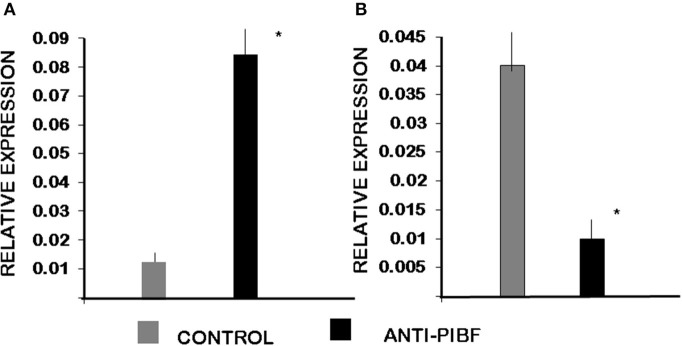
Differential expression of genes involved in Th1/Th2 differentiation by spleen cells of control and anti-PIBF-treated mice. **(A)** IL12A mRNA is significantly upregulated in CD8+ cells of anti-PIBF-treated mice. **(B)** IL-4 mRNA is significantly downregulated in CD4+ cells of anti-PIBF-treated mice. The bars represent the mean ± SEM of four experiments. ^*^P < 0.05.

Among genes involved in the Th1/Th2 pathway, IL-4 was significantly downregulated in CD4+ cells of the anti-PIBF-treated mice. At the same time, IL-12 was upregulated in CD8+ cells of the anti-PIBF-treated animals (Figure 8).

## Discussion

Earlier we showed that PIBF induces the decidual transformation of mouse endometrial stromal cells. Furthermore, PIBF expression in the mouse endometrium is markedly increased during the implantation window (13). These data suggest that PIBF might play an active role in implantation. To confirm this hypothesis, we neutralized the biological activity of PIBF during the peri-implantation period in mice and investigated the consequences of functional PIBF deficiency at several levels.

The anti-PIBF treatment of pregnant mice at days 1.5 and 4.5 of pregnancy resulted in a significantly reduced number of the implantation sites, and the implantations that took place nevertheless must have been compromised as shown by the high resorption rates. In an earlier study, anti-PIBF treatment on day 8.5 of pregnancy increased the resorption rate to 40% (23). The present data show that when administered in the peri-implantation period, the anti-PIBF antibody also interferes with implantation.

Several cell types, e.g., the peripheral pregnancy lymphocytes (24), the embryo itself (25), the trophoblast (26), and the endometrial cells (13), produce PIBF. In confirmed clinical pregnancies, PIBF is detectable in the serum of IVF patients 14 days after embryo transfer (Hudic et al., manuscript in preparation). A single embryo cannot produce such a high amount of PIBF. It is more likely that the bulk of the PIBF is produced by the maternal side in response to the presence of the embryo.

We further investigated the underlying mechanisms of implantation failure and pregnancy loss in functionally PIBF-deficient mice.

Failed pregnancies are characterized by high peripheral NK activity, both in humans and in mice (27–35).

Progesterone decreases the NK activity of peripheral pregnancy lymphocytes in a concentration-dependent fashion (36), and RU 486 (a progesterone receptor antagonist) significantly augments the NK cell cytolytic activity *in vitro* (37).

Decidual NK cells constitute 60% of decidual lymphocytes (38). Is spite of the availability of perforin and granzyme in their cytotoxic granules (38), these cells have a very moderate cytotoxic potential (39, 40) but secrete angiogenic factors and cytokines (38). The dynamics of the appearance of uterine NK cells suggests that one of their functions might be the control of placentation.

The low cytotoxic activity of decidual NK cells might be due to the presence of PIBF in their cytoplasmic granules (22). PIBF blocks the upregulation of perforin expression in activated decidual lymphocytes and inhibits NK cell cytotoxicity by blocking granule exocytosis (21, 41). Bogdan et al. (22) demonstrated a high number of PIBF+ NK cells in the day 12.5 decidua of pregnant mice.

Here we show that anti-PIBF treatment during the peri-implantation period results in the reduced presence of PIBF+ NK cells in the day 10.5 decidua, together with significantly increased decidual and peripheral NK activity, compared to the controls.

Anti-PIBF treatment of mid-pregnant mice has been shown to boost both the peripheral NK activity and the resorption rates. Increased resorption rates in anti-PIBF-treated mice were corrected by simultaneous treatment of the mice with anti-NK antibodies (23), suggesting that PIBF prevents pregnancy loss in mice—at least partly—by blocking NK activity. Increased decidual NK activity owing to the loss of PIBF+ decidual NK cells could be one of the reasons for the increased resorption rates in the anti-PIBF treated mice.

B cells constitute a minor population among decidual lymphocytes, yet they might be important for the immunological balance of the decidua. A recent study showed that the IL-33-induced expression of PIBF1 by decidual B cells protects against preterm labor both in humans and in mice (42).

In the present study, we detected a distinct layer of B cells at the choriodecidual interface of control pregnant mice on day 10.5 of pregnancy. These cells were completely absent from the deciduae of mice that had been treated with anti-PIBF during the peri-implantation period. We could not detect PIBF in the B cells on day 10.5 of pregnancy; however, PIBF+ B cells were present in the late pregnancy deciduae of the control mice (data not shown).

Taken together, it is conceivable that anti-PIBF treatment—by depleting decidual B cells—will, at a later stage, put pregnancy at risk due to the lack of PIBF-positive B cells (42).

Finally, we performed a gene array on the spleen cells of anti-PIBF-treated and control mice in order to investigate whether the absence of functional PIBF had an effect on T cell activation and differentiation.

The T cell receptor is a complex of T cell receptor alpha and beta chains and the CD3 proteins. Activation of CD4+ T cells occurs through the simultaneous engagement of the T cell receptor and a co-stimulatory molecule on the T cell by the MHCII peptide and the co-stimulatory molecules on the APC. In the absence of co-stimulation, T cell receptor signaling results in anergy.

In addition to TCR alpha/beta, a whole set of cell surface receptors are also engaged by their ligands on APCs, which regulate Th differentiation. CD4 acts as a cellular adhesion molecule that binds MHC class II and stabilizes the interaction of T cells and APCs (43, 44). CD28 is a costimulatory receptor on T cells, which binds CD80 and CD86 on activated APCs (45). The TCR alpha/beta/CD3 complex provides the first signal and CD28 the second signal for T cell activation. Both signals are required for IL-2 production and T cell proliferation. CD40 ligand, expressed by activated T cells, binds to CD40 on APCs, initiating a T cell-mediated immune response (46).

In this study, we found that members of the T cell receptor CD3 complex were significantly downregulated on CD4+ T cells of anti-PIBF-treated mice, while CD3D and IL2R B and G were upregulated in CD8+ cells, suggesting that Th cell activation is severely inhibited in the anti-PIBF-treated pregnant mice.

IL-4 is the main cytokine driving Th2 cell differentiation. IL-4 is produced by various cell types, including mast cells, basophils, eosinophils, NK cells, activated CD4+ T cells, and differentiated Th2 cells (47).

Here we found that the gene for IL-4 was significantly downregulated in CD4+ cells, while that of IL-12A was upregulated in CD8+ cells of the anti-PIBF-treated mice.

There is now ample evidence that the recognition of paternal antigens by the maternal immune system is not simply harmless but absolutely necessary for the setting in of the mechanisms that adapt the immune response to tolerate the fetus (48). Following recognition of fetal antigens, the immune system becomes activated, and this will result in the establishment of regulatory mechanisms, e.g., a Th2 dominant immune response (49, 50).

Neutralizing PIBF in the peri-implantation period abolishes this mechanism right at the start. CD4+ T cell activation is disturbed, T cells differentiate in the Th1 direction, and as a result, implantation as well as ongoing pregnancies is compromised.

## Data Availability Statement

The datasets generated for this study are available on request to the corresponding author.

## Ethics Statement

The animal study was reviewed and approved by Animal Care Committee of the University of Pecs.

## Author Contributions

TC performed most of the work. EP and AK did the prime pcr. EM was responsible for the flow cytometry. ZB helped with the animal experiments. This study was designed and supervised by JS-B.

### Conflict of Interest

The authors declare that the research was conducted in the absence of any commercial or financial relationships that could be construed as a potential conflict of interest.
